# Children subjected to family violence: A retrospective study of experiences of trauma-focused treatment

**DOI:** 10.1177/13591045231169147

**Published:** 2023-04-12

**Authors:** Marja Onsjö, Jennifer Strand, Ulf Axberg

**Affiliations:** 1 Department of Psychology, 205656 University of Gothenburg, Gothenburg, Sweden; 2 Faculty of Social Studies, Family Therapy and Systemic Practice, 87368 VID Specialized University, Oslo, Norway

**Keywords:** Trauma, children, child maltreatment, experiences, trauma-focused treatment, family violence, thematic analysis

## Abstract

Exposure to adverse childhood experiences is a risk factor for the development of serious psychiatric and somatic illness. Although trauma-focused therapy is effective in reducing symptoms, not all children benefit from it. To improve treatment efficacy, the children’s perspective on what they perceive as helpful versus hindering is necessary. This study aimed, retrospectively, to explore how children exposed to family violence experienced treatment at the Child and Adolescent Mental Health Service. Seventeen children and youths were interviewed 4–5 years after treatment. The thematic analysis resulted in five themes: confusion, the need to feel heard, fear of consequences, feelings of pain, and identifying oneself as an agent. The results emphasize the importance of the therapeutic relationship, and that trust, genuine interest, and reciprocity are necessary for the child to engage in treatment. However, neither the child’s own agency nor external obstacles such as continuous exposure to abuse should be underestimated in terms of the child’s engagement.

## Introduction

Exposure to trauma such as intimate partner violence and/or abuse during childhood is associated with serious physiological disorders and developmental risks (Anda et al., 2006). Although trauma-focused treatment can effectively reduce trauma-related symptoms, not all children benefit from it (Bisson et al., 2019). To better understand differences in treatment effects, the children’s perspective could be an important source of information. However, studies of the treatment experiences of children subjected to family violence are scarce. To address this knowledge gap, this study aimed, retrospectively, to explore how children exposed to family violence experienced treatment at the Child and Adolescent Mental Health Service (CAMHS).

Adverse experiences may contribute to future serious psychiatric and/or physiological illness (Felitti et al., 1998). Children with experiences of maltreatment are at higher risk of developing behaviour problems, anxiety, depression, post-traumatic stress disorder (PTSD), and suicidal thoughts and behaviours (Gilbert et al., 2009). Exposure to child maltreatment within the attachment relationship is especially harmful (Toth & Manly, 2019), being considered a complex trauma that can cause lifelong problems with attachment security, affect and behaviour regulation, dissociation, and maladaptive cognition (Cook et al., 2003). Furthermore, complex trauma is associated with disturbances in relationships, since it is interpersonal in nature and may affect the possibility of developing trust in, for example, professionals (Mason et al., 2020).

The incidence of multiple traumas is high among patients in and Hultmann and Broberg, 2016 found that up to 50% of participants had experienced child abuse (CA) and/or exposure to interpersonal violence (IPV), and that most had experienced multiple forms of violence. Given the potentially serious consequences of child maltreatment and IPV, effective treatment interventions are crucial.

Research into the effects of psychotherapeutic treatment interventions for children with trauma-related difficulties has increased in recent decades. International guidelines recommend trauma-focused cognitive behavioural therapy (TF-CBT) and eye movement desensitization reprocessing (EMDR) therapy for complex trauma (Bisson et al., 2019). However, studies of trauma-focused treatment after exposure to family violence are still scarce (Gutermann et al., 2016), and previous research has highlighted the need to consider the perspectives of the most vulnerable children (Carter, 2009).

Traditionally, there has been an emphasis on evaluating treatment effects by measuring quantifiable variables such as psychiatric symptoms (Weisz, 2015), but the complexity permeating the life situation of many children in contact with CAMHS also require studies of how therapists meet individual youths’ needs (Ng & Weisz, 2015).

Severe psychiatric symptomatology following exposure to violence within the attachment relationship may require adaptations and treatment interventions tailored to the child’s situation. To better understand how to personalize interventions, children’s subjective experience is important, and recent years have seen growing interest in exploring how children themselves experience CAMHS interventions (Persson et al., 2017). Findings show both positive and negative experiences. Studies indicate that children have difficulties understanding why they are being assessed (Stafford et al., 2016) and experience fear and uncertainty before their first visit (Bone et al., 2014). Other studies of children’s experiences of trauma-focused treatment show that most children appreciated treatment and felt they were offered refuge and validation (Neelakantan et al., 2019), and emphasized the importance of having a therapist they perceive as empathetic, neutral, and expert (Dittmann & Jensen, 2014). The focus on trauma experiences was helpful, although difficult, and the children stressed the importance of interventions being adapted to their own needs, giving them autonomy, control, and choices (Ellinghaus et al., 2021).

This study aimed, retrospectively, to explore how children exposed to family violence experienced treatment at CAMHS. The following research question was investigated: What do children who have received treatment at CAMHS perceive as helpful versus hindering?

## Method

The study included children and adolescents with experiences of family violence who, as CAMHS patients, had participated in a randomized controlled trial comparing TF-CBT with enhanced treatment (eTAU) as usual 4–5 years earlier (Hultmann et al., 2023). In total, 93 children and adolescents participated in the previous study; the present study is based on interviews with 17 of those participants aged 12–25 years (M = 19.8). Ten participants had received TF-CBT and seven eTAU.

### Participants

Seventeen participants were interviewed, 13 female and four males. At the time of the interviews, all had divorced parents and most lived either with their mother (*n* = 9) or father (*n* = 4). One participant lived in a residential home and the remaining three had moved to homes of their own.

Fifteen participants had experienced direct physical violence, 12 psychological violence targeting them, and seven sexual abuse. Experiences of adults in the family abusing drugs or alcohol and/or of witnessing domestic violence were recurrent. Nearly half (*n* = 8) of the participants had made at least one suicide attempt.

Before treatment in the initial study, all participants were assessed for psychiatric symptoms. At that time, 11 of the participants in this study met the diagnostic criteria for PTSD. In addition, some participants met the criteria for other diagnoses: four were diagnosed with depression and/or anxiety, one with attention deficit hyperactivity disorder, and two with oppositional defiant disorder. Five participants did not meet the criteria for any psychiatric diagnosis but exhibited trauma-related symptoms considered clinically relevant and were therefore offered treatment at CAMHS.

### Procedure

Up-to-date contact information was available for 40 of the 93 potential participants from the previous study. One reason why it was difficult to find updated information was that sometimes only parental contact details had been entered in the medical files in the previous study, so no contact information for the children could be found. Another reason was that the participants’ contact information had changed since they were CAMHS patients.

All potential participants were contacted by telephone and given brief information about the study’s aim and procedure. Those who agreed to participate were mailed written information and then contacted again to confirm their interest. Of the 40 potential participants, 17 agreed to participate, 13 declined, and 10 were impossible to reach.

Participants could choose to complete the interviews, which lasted 30 minutes to 2 hours, either at home (*n* = 7) or at CAMHS (*n* = 10).

### Interview

Two clinical psychologists, one of whom is the first author, conducted the interviews. The semi-structured interview guide included 13 questions focusing on participants’ treatment experiences at CAMHS. The questions also concerned their life situation during the treatment period, and experiences of the justice system and the social services, as reported previously (Onsjö et al., 2022). Examples of interview questions and follow-up questions are: “What did you think of the interventions you received at the clinic?”; “Do you remember if you experienced anything in particular as good or bad?”; and “Was there anything you would have liked to change?”

### Analysis

Data were analysed using thematic analysis, a flexible method to identify, analyse, and report patterns of meaning in qualitative data (Braun & Clarke, 2022). A phenomenological (Spinelli, 2005) approach was chosen, as the intention was to capture the participants’ experiences and subjective perspectives. All interviews were recorded and transcribed verbatim, and the analysis followed the six phases described by Braun and Clarke (2006, 2022).

The first author read the transcripts and took note of initial thoughts. Only passages relevant to the research question were thoroughly analysed. The data were systematically analysed and coded inductively. To prevent the influence of the first author’s preconceptions, the process was closely monitored by the other authors. Eight interviews were coded in parallel by the other authors and all codes were compared and discussed. During coding, ideas for themes and assumptions possibly influencing the reading of the data were noted.

Subsequently, the codes were structured into potential themes to find a structure that included as much of the coded data as possible. All authors agreed on the five final themes and their names, which were chosen to capture their essence. Finally, quotations were selected, lightly edited for clarity, and are presented with pseudonyms to preserve participant anonymity.

### Ethics

The study received ethical approval from the regional ethical review board in the designated research area, one of the larger cities in southern Sweden (Dnr: 806-16). Verbal and written information about the study aims and interview procedure was given to all potential participants and, if the children were minors, also to parents or legal guardians. The voluntary nature of participation and the possibility of rejecting questions or terminating participation without explanation or consequences were emphasized. The participants were informed that all data would be anonymized and kept confidential. Written consent was obtained from the participants and, for minors, also from the parents in accordance with legal and ethical guidelines. Furthermore, given the sensitive nature of the study, the interviewers aimed to create an empathetic atmosphere and there was therapeutic readiness to handle reactions to questions about potentially painful areas. When appropriate, participants were also offered psychoeducation on trauma and PTSD during the interview and advised on how to seek treatment for trauma-related problems. To our knowledge, at least one participant was motivated to contact school healthcare following the interview.

### Reflexivity

The first author is one of the therapists who conducted treatments in the previous randomized treatment study. Based on this, there was researcher experience of trauma-focused treatment being helpful, as well as awareness that the therapeutic setting does not always succeed in meting each child’s needs. In trying to meet the needs of children with experiences of family violence and to better understand what makes treatment meaningful to them, a preconception was that the children’s perspective often was missing.

In all cases where the first author had been involved in the previous treatment interventions, the interviews were conducted by the other clinical psychologist. During all study phases, there was awareness of the first author’s preconceptions, which were handled by challenging discussions, self-reflection, and supervision.

## Results

Participants generally described their experiences of treatment at CAMHS as positive and many mentioned being met with kindness and feeling heard by the therapists. Some described the therapeutic work as helpful in improving their wellbeing, some said that their relationship with a parent had strengthened, and others even described the treatment as life-changing. However, many also identified areas for improvement structured around these five themes: confusion, the need to feel heard, fear of consequences, feelings of pain, and identifying oneself as an agent.

### Confusion

An ongoing theme was confusion over the contact with CAMHS. Confusion permeated the overall situation: the referral process, the treatment purpose, the diagnosis, specific parts of the treatment intervention, and the reason why the treatment ended.

Confusion about the referral concerned the fact that few had initiated contact with the clinic themselves; rather, most had been referred by professionals or taken there by a parent. Earlier experiences of social services or healthcare made it difficult to understand the aim of the different agencies. Many described coming to the clinic with only a vague idea of having to talk to someone and not knowing what was expected. Several described participating in meeting after meeting, answering question after question, with no understanding of how these procedures were supposed to help:

They just gave me piles and piles of paper … I never really understood what I was supposed to get out of it because they still didn’t help me after I had answered in all these papers. (Amelie)

Receiving a diagnosis was sometimes a relief in that it helped them understand their reactions, but most described being diagnosed in negative terms. They expressed confusion and a sense of injustice at being defined as the problem when someone else had been violent towards them:

It was kind of difficult – all these different concepts – and then I was the one classified as having problems. Because I wasn’t the one who had done anything wrong. (Tanja)

The concept of treatment was hard to understand for many. Although they said that it had been a relief to talk to someone, the aim of the treatment had been unclear:

I don’t think I really understood the purpose. I just thought, okay, this is treatment, this is going to help, but I don’t think I ever really understood. The only thing I needed was to get help, to talk to someone … I’m not sure if I really grasped the actual treatment. (Tanja)

Components of specific treatment interventions were often unclear. For example, participants had been puzzled about the purpose of writing down traumatic events in TF-CBT or watching the therapist’s moving fingers while thinking of various memories in EMDR. Few, however, had questioned the interventions. Olivia described pretending that the intervention was successful out of concern for the therapist’s feelings:

It was kind of embarrassing because I didn’t feel that [laughs] anything happened. I never had any thoughts or anything, so I remember that I lied, I said “Ah, I can feel it, I can see it.” (Olivia)

Tanja said she had appreciated going to CAMHS, but she had never understood why the treatment ended when her family still had severe problems. She wished that someone had contacted them to ask if they needed more help.

### The need to feel heard

A common theme in the interviews was the importance of feeling heard. Therapists who were perceived as empathetic, treated participants as individuals, let them participate in treatment planning, and confirmed their pain contributed to the sense of being heard.

Being treated with empathy was described as important by many and, with few exceptions, the therapists had been perceived as patient and friendly. Being heard concerning the need to see the same therapist was a precondition for some to feel safe enough to engage in therapy:

For me it had to be the same person. That was something that my mother made sure of, that it was the same one, otherwise it wouldn’t have worked, because I had great difficulty trusting others – which is understandable. (Terese)

For others, being able to replace a therapist they did not feel connected to allowed them to give therapy a second chance. Most wanted a therapist perceived as a genuine listener who would give them time and space to express their own views of their situations and problems. Vega, for instance, said that the only thing she wanted was to reconnect with her siblings, which the therapist was unable to help her with, but the fact that the therapist listened to her and confirmed her feelings had motivated her. Some also described how therapists who expressed preconceived ideas about their problems had made them feel reluctant to engage in treatment. Amelie expressed anger at therapists she felt had dismissed her story:

Even if they’ve heard it all before, they shouldn’t say like “Yes okay, but I already know” or “I’ve heard this before” … . They just shouldn’t be so fucking high up in their own heads. (Amelie)

One aspect of feeling heard was that many had appreciated being asked how they preferred to learn new things and had been offered choices by the therapist. Role playing, drawing on a whiteboard, using dolls to re-enact events, or writing a book with the help of the therapist were mentioned as positive alternatives to just talking. Tyra described the importance of being active and able to try new ways of acting:

I remember that I never got bored because it was not like we talked all the time. It was more like there were dolls, there was a theatre, there was sand. It was like, what could I do when I got so angry and when I didn’t know what to do? [There were] things that I could try out. (Tyra)

However, being asked to participate in decisions and in planning the work was not straightforward. Making decisions about things such as where they wanted to start or how they wanted to do an exercise was perceived as positive, but some felt relieved at not having to make decisions about the treatment itself.

Most participants stressed the importance of children being able to disclose abuse or other traumas to an empathetic listener, and felt that talking about their experiences had been a way to heal. As Liv said, “It’s good – you need to talk about things that are painful.” They also emphasized the importance of the therapist’s understanding of how painful and challenging it could be and their appreciation of being met with kindness and patience. Many appreciated being given candy or little rewards, confirming that the therapist acknowledged their effort and understood their pain. At the same time, a few participants felt that the focus on violence prevented them from talking about other things that had been occupying them:

What was going on at home at the moment, my relationships with friends, things like that. Girls I might be interested in, that sort of thing – you never got to it. That is what I kind of think is so hard in going to CAMHS, I never got to talk about what I really wanted to talk about. (Harald)

### Fear of consequences

This theme captures the participants’ fears of potential consequences if they disclosed that they were still experiencing physical and psychological violence and abuse. These fears had sometimes prevented them from engaging in treatment, whereas a more proactive approach from the therapist might have facilitated the process.

Continuous exposure to abuse and a need to keep it secret from the therapists made it difficult to engage in treatment: siblings had violently controlled the family, and parents had handed out punishments after CAMHS meetings and were continuously threatening. Fear of the consequences a disclosure might have, of the family turning its back on them, or of social services being contacted made it impossible to be open about the situation:

As soon as I came home I noticed that Dad had a bad attitude because I had been at a meeting. Now that I’m older, it is clear to me why I cancelled the meetings. There were penalties and my pocket money was withdrawn. (Irma)

For some, it was clear that the secrets they kept had hindered the therapy’s success. For example, Olivia had been sexually abused but never disclosed it to the therapist. She had said that she had a secret, but this was not further addressed by the therapist, whom she felt almost made a joke out of it. In retrospect, she would have wanted the therapist to be more active in posing questions and in clarifying that she would never blame Olivia:

If she had asked me more about it, maybe I would have dared to tell. If I had known that one is never responsible for being put through such things and that there is nothing to be ashamed of. (Olivia)

### Feelings of pain

This theme concerns the painful emotions that the experiences of violence had caused the participants: feeling broken, ashamed, and different from others. Identifying as a patient could reinforce feelings of alienation, as the focus on violence in treatment was often distressing and the purpose of the therapy could be incomprehensible. Parents’ participation in treatment was appreciated overall but was often stressful, with experiences of joint sessions ranging from highly positive to disappointing or disillusioning.

The pain of feeling different from others was a recurring theme. Many described how their self-image had been negatively affected by the violence and that they felt ashamed, disgusting, and broken. They also blamed themselves for being sexually abused or for being unable to prevent violence against themselves or others. Some depicted themselves as stupid or crazy, both as victims of abuse and for the reactions they suffered afterwards:

After everything I’d undergone, I got an injury in my head … I thought about it all the time, my mind was always occupied with it and so I … everything was chaotic, I caused a lot of problems. (Patricia)

Being a patient and being diagnosed sometimes reinforced the children’s distress at feeling different, and the treatment setting made some feel even more alienated. A few mentioned that they would rather have met in a less institutional environment, to make the conversation feel more normal: “It would feel better because you wouldn’t have the same view of yourself as being different” (Otto).

Talking about experiences of violence and abuse had been challenging, causing many to re-experience the pain. Participants’ understandings of the purpose of therapy differed, and some said that re-experiencing the pain was part of a process that made the pain understandable, and that the pain decreased as the work progressed:

It was kind of like I experienced it again, so it was really hard for me. But over time I learned to be able to talk about it without being as affected, so it’s really good now. (Vega)

Others never understood why they had to talk about the events repeatedly and felt no relief from re-experiencing the pain. Amelie described how talking about her memories of the violence made her experience severe anxiety. In retrospect, she said, she needed a more hands-on approach from the therapists, whom she wished had taken charge of the conversation and helped her regulate her reactions:

Most of the time it was up to me to decide what we would talk about … . This was both good and bad at the same time, because I went for the worst parts directly and it was very difficult for me to talk about them … and the psychologists were unable to calm me down. (Amelie)

Sometimes a participant would read a narrative about the abuse to the accompanying parent. This was a stressful undertaking that, depending on the parent’s reaction, ended in different ways. Sometimes it led to a closer and stronger relationship, as it did for Marianne, who said that it improved her later communication with her mother:

It was very scary and I remember I couldn’t even look up from the paper … once you’ve done it, that’s when you start to feel free … it keeps feeling better and better. (Marianne)

Annelie, in contrast, described having repeatedly stated her unwillingness to share her narrative with her mother, but felt pressured to go through with it. The therapist was unaware of the unsafe family situation and Annelie described how she had come to treatment with her bag packed, fearing that her mother would kick her out when she heard what she had disclosed in therapy:

That was the hardest part – I was able to talk about it and write the story, but reading it out loud to my mother, I think that was the hardest thing I have done. (Annelie)

Some parents who had let participants down by abandoning them, not taking responsibility for the violence, or being continuously threatening had been invited to meetings. Participants were unsure why, but guessed there had been a hope of changing the parent’s attitude. These joint meetings left many feeling disappointed and disillusioned.

### Identifying oneself as an agent

This theme captures how some participants repeatedly identified themselves as agents: their attitude and motivation had been decisive for the treatment outcome. The characteristics of the therapist were less important; instead age, other psychiatric symptoms, and ongoing substance abuse were cited as examples of factors that affected their engagement.

Throughout the interviews, the participants highlighted their own attitude and motivation as the most important factors affecting whether or not treatment had been successful. They identified as agents. Some described being reluctant at first but changing their minds once they started to trust the therapist:

I didn’t believe it could help, I didn’t want to be there. But then, when I had been there a couple of times, I noticed how I opened up more and more. (Vega)

For those who were motivated from the beginning, the therapist was less important; they had decided that therapy was going to help them and were determined to go through with it no matter what. Nadja described having to change therapists. Although she did not like her new therapist as much as her first, she had decided to not let it hinder her: “Fuck it … I’ll just do it anyway!”

Others said that all efforts of the therapists were wasted since they had entered the situation determined not to participate:

Right from the beginning I felt that this wasn’t going to be very helpful for me, it wouldn’t provide me with anything …. Since I was reluctant to receive any help, it might have made it difficult to help me. (Artur)

Age-related personal characteristics were described as having hindered the success of therapy for some, such as Patricia who described being too young and impatient at the time: “They couldn’t have made it any clearer – it was me, I just closed my ears.” Ongoing substance abuse, an eating disorder, or other psychiatric symptoms that the participants did not feel confident or motivated enough to disclose to the therapist also made it hard to engage in treatment.

## Discussion

The study aimed, retrospectively, to explore how children exposed to family violence experienced treatment at CAMHS.

Five themes were identified: confusion, the need to feel heard, feelings of continued fear, feelings of pain, and the importance of agency. These themes are not disconnected but interact with and amplify each other. This discussion begins by considering how the themes and results fit into existing research and ends with an outline of their clinical implications.

Before discussing the different themes, it is important to emphasize that the participants generally described their experiences of treatment as positive and most stressed the importance of facing hurtful memories. Relational aspects were highlighted, and many were persuaded to engage in treatment by the therapist’s kindness, empathy, and genuine interest.

Many participants experienced the period of treatment at the CAMHS as confusing, as captured by the first theme. This bewilderment permeated the overall situation: the referral process, the treatment purpose, the diagnosis, specific parts of the treatment intervention, and the reason why the treatment ended. The confusion expressed by the participants aligns with previous research (Dittman & Jensen, 2014) finding that children have difficulties understanding why certain questions and assignments are part of therapy, emphasizing the importance of clarifying why and how the treatment will be performed. Also, the uncertainty about what to expect and the nervousness reported by our participants are aligned with the findings of Bone et al. (2014), who advocated for improved information for the family before the first visit.

Another source of confusion was uncertainty about the treatment goal and specific treatment components. Some said that it had been difficult to understand how “all the talking” was supposed to help, and some had preferred alternatives such as playing, painting, and writing. Feeling that the therapist was trying to explore their individual learning styles and adjust the interventions accordingly was motivating. This aspect of treatment is arguably even more important for younger patients, as children may require information and ways of working adapted to their age and maturity.

Nevertheless, as captured by the second theme, several participants had been willing to follow instructions even though their purpose was unclear. A precondition for their engagement and compliance was regarding the therapist as an empathetic listener genuinely interested in their situation and in them as individuals. This interest gained their trust and fostered what could be seen as a “real relationship” characterized by the perception of the therapist as warm, caring, understanding, and curious (Wampold, 2017). Accordingly, therapists perceived as preoccupied with their own agenda had reduced some participants’ willingness to engage. Several participants received manualized evidence-based treatment. This can ensure effectiveness but risks creating situations where the patient’s background and individual life situation are overlooked (Kazdin, 2017), or risks hampering the therapist’s ability to be open and flexible in approaching the complexity of the child’s individual needs (Weisz et al., 2019). Other research, however, indicates that using a manual in trauma-focused treatment does not compromise the formation of a positive therapeutic relationship (Ormhaug et al., 2014).

Another aspect that affected the participants’ engagement, as emphasized in the third theme, was fear of the consequences of disclosing that they were still experiencing physical and psychological violence and abuse. Some parents were resistant to the child having contact with CAMHS and even punished the child for attending. These findings are supported by a previous study showing that children exposed to family violence risk additional violence and other potential traumas while receiving care (Onsjö et al., 2022).

Several participants reported that a parent had struggled with mental illness, and that this had affected them. This is in line with the finding of Campbell et al. (2021) showing that children suffering from mental illness frequently have a parent who is mentally ill, which might affect these children’s recovery. Furthermore, Birmes et al., (2009) identified the importance of also addressing and assessing parental mental health before treating the child, since it tends to affect both the development of the child’s trauma symptoms and the effectiveness of treatment.

Involving parents in treatment also helps improve the parent–child relationship and reduces parents’ feelings of distress, shame, and guilt (Mastorakos et al., 2021). However, the fact that some participants experienced violence and abuse while in treatment at CAMHS highlights the importance of not assuming that a child is protected and of recognizing that ongoing violence might be detrimental to the child’s engagement in therapy. It is crucial to routinely address the child’s safety and explore whether a child’s reluctance to have a parent join in the treatment might be due to fear of retribution.

Pain and distress were frequently evident in participants’ descriptions of how the violence had affected them, as highlighted in the fourth theme. Many described having a negative self-image, sometimes reinforced by being a patient at CAMHS, and said that the treatment’s focus on violence was painful. Although not all participants experienced the treatment as helpful, most stressed the importance of disclosing experiences of violence. Again, relational aspects such as perceiving the therapist as genuinely caring were preconditions for effective treatment. Some mentioned that it was important for the therapist to notice their distress and recognize their hard work; others felt encouraged by receiving small rewards and candy. Research has shown that talking about painful experiences is both crucial to recovery as well as distressing and difficult (Dittmann & Jensen, 2014; Eastwood et al., 2021). Given the stressful nature of such therapy and the fact that the patients are children, encouragement through small rewards and treats could play an important role in increasing their engagement.

Throughout the treatment, the participants depicted themselves as active in relation to the care received and as having their own agency, making their attitude decisive for the treatment outcome. This last theme – identifying oneself as an agent – is in line with the importance of mutual agreement on the tasks and goals of therapy and of the patient’s active engagement in behaviours that promote positive change. Additionally, the patient needs to believe that the therapy is meaningful and to view progress as resulting from their own efforts (Wampold, 2017). Given the importance the participants placed on their own engagement, it is noteworthy that some children who were initially unmotivated to participate changed their minds when the therapist was genuinely interested. Trauma-focused treatment has been found to be more effective when youths experience the therapeutic relationship positively (Ovenstad et al., 2021), as seen in our participants’ emphasis on the importance of the therapists demonstrating empathy. However, as the consequences of childhood exposure to violence are serious, it is important to ensure that diverse interventions aimed at increasing safety and preventing violence are offered by, for example, schools, social services, and community-based organizations in addition to CAMHS.

### Clinical implications

In this study, the children’s experiences of treatment were dependent on relational aspects. A prerequisite for facing painful experiences was a therapist who was empathetic, patient, and genuinely interested. This requires that therapists are given organizational conditions such as time and room to be creative, regardless of the specific treatment methods.

The children were agents with agendas of their own, not passive recipients or treatment. Their motivation and commitment to therapy first became established when they felt respected and treated as individuals, not as problems to be solved. It seems essential to provide a setting characterized by patience and space for the children to share their perspectives on what might help them. This might be even more important when the children have experienced family violence, which may have affected their ability to trust adults.

Finally, it is important to remember that children will likely continue to experience insecurity during treatment. Routinely asking about ongoing violence or abuse and creating safety plans when indicated should be part of standard practice.

### Strengths and limitations

The study had a retrospective design and the participants were interviewed several years after their treatment. The experiences shared here are therefore based on memories rather than verifiable accounts. As time passes, memories tend to fade and events are reinterpreted, which might have affected the results. However, the time elapsed could also have been a strength. Several participants revealed information about how insecure their home and life situations had been during treatment, information that they had not felt safe enough to disclose during treatment. Their capacity to verbalize and reflect on their situation at the time of treatment may have increased with their growing maturity.

This study offers insight into the experiences of a limited group of participants, so its findings may not be representative of all participants in the previous study. However, the phenomenological design gives comprehensive insight into the personal narratives and experiences of the participants. Furthermore, the findings highlight the complexity of the life situations of children subjected to violence, indicating that further studies are necessary to explore how inter-agency collaboration can foster children’s safety and mental health.
